# Generating Daily Soil Moisture at 16 m Spatial Resolution Using a Spatiotemporal Fusion Model and Modified Perpendicular Drought Index

**DOI:** 10.3390/s22145366

**Published:** 2022-07-19

**Authors:** Xin Lu, Hongli Zhao, Yanyan Huang, Shuangmei Liu, Zelong Ma, Yunzhong Jiang, Wei Zhang, Chuan Zhao

**Affiliations:** 1 Sichuan Research Institute of Water Conservancy, Chengdu 610072, China; scsky_lx@126.com (X.L.); sky_liushuangmei@sina.com (S.L.); sky_mazelong@163.com (Z.M.); zhaochuanvip@163.com (C.Z.); 2 Department of Water Resources, China Institute of Water Resources and Hydropower Research, Beijing 100038, China; zhaohl@iwhr.com (H.Z.); lark@iwhr.com (Y.J.); 3 School of Software Engineering, Chengdu University of Information Technology, Chengdu 610200, China; 4 China Electronics Technology Group Corporation (CETC), Big Data Research Institute Chengdu Branch Co., Ltd., Chengdu 610093, China; zhang106wei@sina.com; 5 National Engineering Laboratory for Big Data Application on Improving Government Governance Capabilities, Guiyang 550081, China

**Keywords:** remote sensing, soil moisture (SM), physical model, spatial downscaling, modified perpendicular drought index (MPDI), European Space Agency’s Climate Change Initiative (ESA CCI), water resources management

## Abstract

Soil moisture (SM) is an important parameter in land surface processes and the global water cycle. Remote sensing technologies are widely used to produce global-scale SM products (e.g., European Space Agency’s Climate Change Initiative (ESA CCI)). However, the current spatial resolutions of such products are low (e.g., >3 km). In recent years, using auxiliary data to downscale the spatial resolutions of SM products has been a hot research topic in the remote sensing research area. A new method, which spatially downscalesan SM product to generate a daily SM dataset at a 16 m spatial resolution based on a spatiotemporal fusion model (STFM) and modified perpendicular drought index (MPDI), was proposed in this paper. (1) First, a daily surface reflectance dataset with a 16 m spatial resolution was produced based on an STFM. (2) Then, a spatial scale conversion factor (SSCF) dataset was obtained by an MPDI dataset, which was calculated based on the dataset fused in the first step. (3) Third, a downscaled daily SM product with a 16 m spatial resolution was generated by combining the SSCF dataset and the original SM product. Five cities in southern Hebei Province were selected as study areas. Two 16 m GF6 images and nine 500 m MOD09GA images were used as auxiliary data to downscale a timeseries 25 km CCI SM dataset for nine dates from May to June 2019. A total of 151 in situ SM observations collected on 1 May, 21 May, 1 June, and 11 June were used for verification. The results indicated that the downscaled SM data with a 16 m spatial resolution had higher correlation coefficients and lower RMSE values compared with the original CCI SM data. The correlation coefficients between the downscaled SM data and in situ data ranged from 0.45 to 0.67 versus 0.33 to 0.54 for the original CCI SM data; the RMSE values ranged from 0.023 to 0.031 cm^3^/cm^3^ versus 0.027 to 0.032 cm^3^/cm^3^ for the original CCI SM data. The findings described in this paper can ensure effective farmland management and other practical production applications.

## 1. Introduction

Traditional soil moisture (SM) monitoring schemes are mainly based on data collected at in situ sites. Some techniques have been developed to measure SM with ground instruments, for example, gravimetric methods [1], time domain reflectometry [2], capacitance sensors [3], and electrical resistivity measurements [4]. These data have the advantage of high authenticity. However, the disadvantages of these schemes include high costs and the accuracy can be easily affected by the sites’ distribution. The spatial representativeness of discrete sites are limited to some extent at the regional scale. Remote sensing technologies can effectively compensate for the deficiencies in traditional monitoring methods [5,6,7]. Remotely sensed data have become an important data source for SM monitoring because of their objectivity, low costs, large scopes, and continuity [8,9]. A variety of global-scale SM products (e.g., the Advanced Microwave Scanning Radiometer–EOS (AMSR-E) Land Parameter Retrieval Model (LPRM) [10], advanced scatterometer (ASCAT) [11], Soil Moisture and Ocean Salinity(SMOS) [12], and European Space Agency’s Climate Change Initiative (ESA CCI) SM products [13]) are available to support large-scale applications [6]. However, the low spatial resolutions of these products introduce difficulties in meeting the needs of small- and medium-scale applications. Therefore, a variety of spatially downscaling methods have been proposed [14], such as (1) empirical-model-based or polynomial fitting methods [15,16], which express the high-resolution SM as a polynomial function of land surface temperature (LST), vegetation index, and surface albedo derived from optical/thermal data; (2) physical-model-based or evaporation-based methods [17,18], which are based on the soil evaporation process to link optical and near-surface SM data; and (3) machine-learning-based methods [19,20].

**Figure 1:**
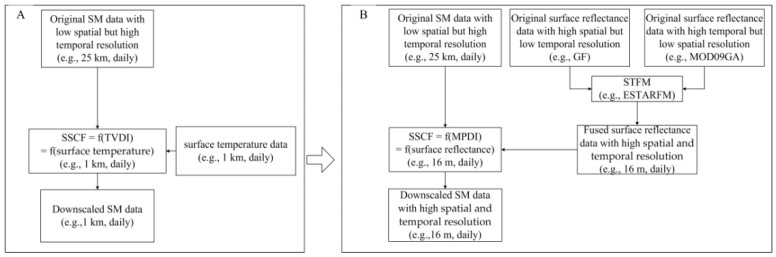
Overview comparisons between the traditional spatially downscaling method with surface temperature data as auxiliary parameters (**A**) and the proposed spatially downscaling method with fused surface reflectance data as auxiliary parameters (**B**).

Currently, physical-model-based methods are widely used. The core concept of these methods involves establishing spatial scale conversion factors(SSCFs) between different spatial scales. From the original Merlin method (i.e., disaggregation based on physical and theoretical scale changes (DISPATCH)) [17], conversion factors based on the soil wetness index (University of California at Los Angeles (UCLA)-SWI) [21], vegetation temperature condition index (UCLA-VTCI) [22], and temperature vegetation drought index (Peking University (PKU)-TVDI) [18] have been developed successively. However, the SSCFs mentioned above require surface temperature products as auxiliary parameters (Figure 1A). The low spatial resolutions (e.g., >1 km) of surface temperature data generally limit the degree of spatial refinement that can be achieved by such downscaling methods. The scope of application of remotely sensed SM data will be limited, especially in a complex surface area where small plots are distributed (e.g., <16 m × 16 m). Products obtained in the optical/near-infrared band usually have higher spatial resolutions (e.g., <30 m, such as the Landsat Thematic Mapper (TM) or Gaofen 6 (GF6) products). Additionally, good correlations have been found between some drought indexes constructed by surface reflectance bands (e.g., modified perpendicular drought index (MPDI) values) and the corresponding SM values [23,24]. Therefore, if an SSCF is constructed by using a kind of drought indexes, a spatially downscaled SM product with a higher spatial resolution (e.g., 16 m) is expected as a result. However, a new problem of low temporal resolution might be brought, which may lead to the high-temporal-resolution feature of the original SM data that cannot be maintained, if surface reflectance data are used for downscaling, because the temporal resolutions of surface reflectance data are usually low (e.g., Landsat TM: 16 d). Coupled with frequent cloud disturbances [25], the effective observation frequency of these data is further reduced. In recent years, to solve this “*spatiotemporal contradiction*”, many spatiotemporal data fusion algorithms have been proposed, including (1) spatial and temporal adaptive reflectance fusion model (STARFM) series algorithms [26,27], (2) unmixing-based algorithms [28,29], (3) sparse representation series algorithms [30,31], and (4) machine learning algorithms [32]. In this paper, the enhanced spatial and temporal adaptive reflectance fusion model (ESTARFM), which is widely used, was selected to construct a surface reflectance dataset with high spatial and temporal resolution (e.g., 16 m, daily). Then, the surface reflectance dataset can be further used to generate an MPDI dataset and an SSCF dataset for downscaling (Figure 1B).

In summary, this paper proposes the following research ideas for downscaling the original SM products. First, nine 16 m surface reflectance datasets were generated based on the ESTARFM algorithm by fusing two 16 m GF6 images and nine 500 m MOD09GA images. Second, nine 16 m SSCF images were calculated based on an MPDI dataset and an aggregated MPDI dataset, constructed by the surface reflectance dataset. Finally, nine downscaled CCI SM images with a 16 m spatial resolution were obtained based on the SSCF images and the original CCI SM images. This paper took five cities in southern Hebei Province as study areas. Then two GF6 images, nine MOD09GA images, and nine corresponding ESA CCI images in study areas were collected for research data from 1 May to 11 June 2019. A total of 151 in situ SM data were collected on 1 May, 21 May, 1 June, and 11 June, which were used for the quantitative evaluations of the proposed method.

## 2. Materials and Methods

### 2.1. Study Area

**Figure 2:**
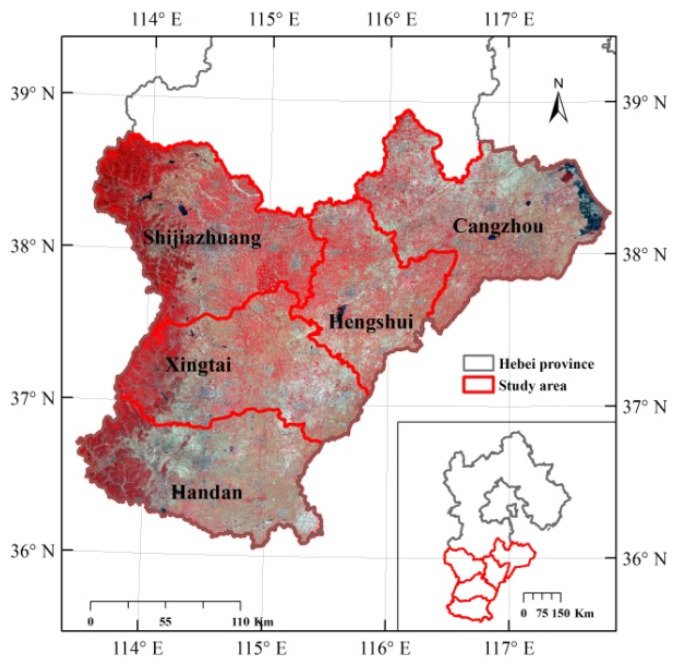
Geographic location and NIR-red-green composite of a GF image of the study area taken on 3 June 2019.

In this paper, five cities (i.e., Shijiazhuang, Xingtai, Handan, Cangzhou, and Hengshui) in southern Hebei Province were taken as the research area (Figure 2). Hebei Province is a large agricultural province in China. In this region, SM monitoring is critical for predicting agricultural growth and controlling water conservation. The study area belongs to the North China Plain and has a temperate continental monsoon climate with four distinct seasons. The elevation ranges from 0 to 2268 m, the average elevation is 140 m, and the study area is approximately 61,100 km^2^.

### 2.2. Datasets and Preprocessing

#### 2.2.1. GF and MODIS Reflectance

Two 16 m GF6 images and 9500 m MOD09GA images were selected as the ESTARFM algorithm input data (Table 1). GF images were acquired on 2 May and 3 June 2019. MOD09GA images were acquired on 1 May, 2 May, 20 May, 21 May, 22 May, 23 May, 28 May, 1 June, and 11 June 2019. Since each GF reflectance band corresponds to a MODIS band with a similar spectral width, GF and MODIS data products contain 4 corresponding bands. All 4 of these bands (i.e., red, green, blue, and near-infrared) were used in the ESTARFM algorithm, although the MPDI values were only calculated using red and near-infrared bands.

#### 2.2.2. ESA CCI SM

The European Space Agency (ESA) released the first multidecade global-scale satellite-derived SM dataset in 2012 as part of the CCI project. The ESA CCI dataset incorporated microwave data and had the longest available SM data series. The ESA CCI SM product (with a spatial resolution of approximately 25 km × 25 km) was established as a daily SM product with global coverage. The combined CCI SM data (Version 4.5) were used for the downscaling process described in this study. Nine CCI SM images were selected with collection times corresponding to those of the MOD09GA product (Table 1).

#### 2.2.3. In Situ SM Data

In situ SM data were collected during the growing period from March to November on the 1st, 11th, and 21st day of each month. In situ SM data, measured by drying–weighting method, were taken at a depth of 10 cm. SM sensors (i.e., TRIME-EZ/IT, IMKO, Ettlingen, Germany), installed at the flux tower, provide daily SM as volumetric moisture content (cm^3^/cm^3^) at different depths of 10, 20, 40, 80, and 100 cm. Normally, the SM products provided by microwave remote sensing are considered to represent the surface SM (the top 5–10 cm of the soil). Therefore, the SM measured at 10 cm was used in our study to investigate the performance of satellite-derived SM. Additionally, all the selected sites were in local areas with relatively uniform local feature classifications and conditions. A total of 151 SM monitoring sites were measured on 1 May, 21 May, 1 June, and 11 June. Specifically, 37 sites were sampled on 1 May, 36 sites on 21 May, 37 sites on 1 June, and 41 sites on 11 June.

### 2.3. Downscaling Approaches

#### 2.3.1. Research Flowchart

**Figure 3:**
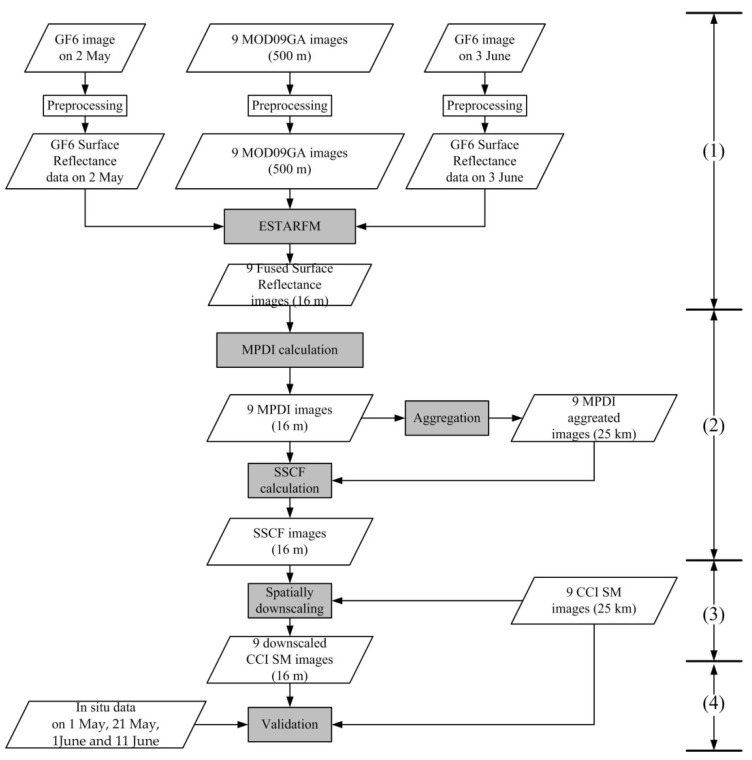
Research flowchart of the proposed method.

A flowchart displaying the research process proposed in this paper is shown in Figure 3. (1) First, based on the ESTARFM algorithm, a surface reflectance dataset with high spatial and temporal resolution (a total of 9 periods, 16 m spatial resolution) was constructed by fusing 2 GF6 images and 9 MOD09GA images. (2) Second, based on the fused dataset, an MPDI dataset (a total of 9 phases, 16 m spatial resolution) was calculated. Additionally, an SSCF dataset (a total of 9 periods, 16 m spatial resolution) was calculated by the MPDI dataset and the aggregated MPDI dataset (a total of 9 phases, 25 km spatial resolution). (3) Third, the CCI SM dataset was downscaled from 25 km to 16 m (a total of 9 phases) by the SSCF dataset, reference to the spatially downscaling formula described in detail in Section 2.3.2. (4) Finally, the original CCI SM images and in situ SM observations were used to verify the accuracy of the downscaled results.

#### 2.3.2. Core Algorithms

##### ESTARFM

Gao et al. [26] proposed the STARFM based on Landsat and MODIS data, which can be fused to generate images that are highly consistent with real conditions [26,33]. The results of a few experimental studies confirmed that the STARFM algorithm has a relatively poor synthesis effect when no low-spatial-resolution pure pixels are present in the sliding window for a region with high spatial heterogeneity. The ESTARFM algorithm solves this problem to some extent by introducing the endmember conversion coefficient [27].

For regions with high spatial consistency, most low-spatial-resolution MODIS pixels are mixed pixels. According to the linear spectral mixing model, the reflectance of a mixed pixel is assumed to be equal to the sum of the weights of the reflectance of the ground objects within the pixel range and their corresponding proportion of the pixel area:

(1) $$M\left(x_{i},y_{j},t_{k}\right)=\overset{m}{\underset{n=1}{\sum }}f_{n}*G\left(x_{i},y_{j},t_{k}\right)+\varepsilon_{0}$$

(2) $$M\left(x_{i},y_{j},t_{l}\right)=\overset{m}{\underset{n=1}{\sum }}f_{n}*G\left(x_{i},y_{j},t_{l}\right)+\varepsilon_{0}$$

where *M* (*x_i_*, *y_j_*,*t_k_*) and *G* (*x_i_*, *y_j_*,*t_k_*) indicate the surface reflectance at a given pixel location (*x_i_*, *y_j_*) in a MODIS image or GF image at *t_k_*,ε_0_ indicates the difference between the MODIS-derived surface reflectance and the GF-derived surface reflectance caused by different bandwidths and solar geometries, *f_n_* denotes the proportion of the surface area occupied by the *n*-th class, and *f_i_* stays constant from *t_k_* to *t_l_*. According to Equations (1) and (2), we can obtain the following formula:

(3) $$M\left(x_{i},y_{j},t_{k}\right)-M\left(x_{i},y_{j},t_{l}\right)=f_{n}*\overset{m}{\underset{n=1}{\sum }}(G_{i}\left(x_{i},y_{j},t_{k}\right)-G_{i}\left(x_{i},y_{j},t_{l}\right))$$

The surface reflectance of the *n*-th class is assumed to change linearly from *t_k_* to *t_l_*, as follows:

(4) $$G_{n}\left(x_{i},y_{j},t_{k}\right)-G_{n}\left(x_{i},y_{j},t_{l}\right)=r_{n}*\Delta t$$

where *r_n_* is the change rate of the surface reflectance of the *n*-th class. By substituting Equation (4) into Equation (3), the following equation can be obtained:

(5) $$M\left(x_{i},y_{j},t_{k}\right)-M\left(x_{i},y_{j},t_{l}\right)=\Delta t*f_{n}*\overset{m}{\underset{i=1}{\sum }}r_{n}.$$

In Equation (5), the surface reflectance of the *n*-th class in the GF image can be known at *t_k_* and *t_l_*, and the following formula can be obtained:

(6) $$\Delta t=(G_{n}\left(x_{i},y_{j},t_{k}\right)-G_{n}\left(x_{i},y_{j},t_{l}\right))/r_{n}$$

By substituting Equation (6) into Equation (5), the following expression is obtained:

(7) $$\frac{G_{n}\left(x_{i},y_{j},t_{k}\right)-G_{n}\left(x_{i},y_{j},t_{l}\right)}{M\left(x_{i},y_{j},t_{k}\right)-M\left(x_{i},y_{j},t_{l}\right)}=\frac{r_{n}}{f_{n}*\sum_{i=1}^{m}r_{n}}=v_{n}.$$

where *v_n_* denotes the ratio of the change rate of the *n*-th class to the change rate of the whole mixed pixel from *t_k_* to *t_l_*, *v_n_* is a constant, and *f_n_* and *r_n_* are constants. The surface reflectance of the GF image can be obtained from Equation (7) at any time, *t*_0_, between *t_k_* and *t_l_*:

(8) $$G_{n}\left(x_{i},y_{j},t_{0}\right)=G_{n}\left(x_{i},y_{j},t_{k}\right)+v_{n}*\left(M\left(x_{i},y_{j},t_{k}\right)-M\left(x_{i},y_{j},t_{l}\right)\right)$$

When the algorithm is implemented, the endmember conversion coefficient of the *n*-th class (i.e., *v_n_*) can be calculated using Equation (7) based on the surface reflectance images taken at *t_k_* and *t_l_*. According to Equation (8), the surface reflectance of the *n*-th class can be obtained at any time *t*_0_ (i.e., *G_n_* (*x_i_*, *y_j_*, *t*_0_)) between *t_k_* and *t*. In consideration of the mixed-pixel effect, land cover and phenological changes, and solar geometry-related bidirectional reflectance distribution function (BRDF), by introducing information representing similar neighboring pixels in a given search window, the unknown Landsat reflectance of the center pixel (*x_ω_*_/2_, *y_ω_*_/2_) at *t*_0_ can be expressed as follows:

(9) $$\begin{array}{ll} G_{n}\left(x_{\omega /2},y_{\omega /2},t_{0}\right) & \\ & =G_{n}\left(x_{\omega /2},y_{\omega /2},t_{0}\right)+v_{n}\\ & *\overset{\omega }{\underset{i=1}{\sum }}\overset{\omega }{\underset{j=1}{\sum }}\overset{\alpha }{\underset{k=1}{\sum }}W_{ijk}*\left(M\left(x_{i},y_{j},t_{k}\right)-M\left(x_{i},y_{j},t_{l}\right)\right) \end{array}$$

where *ω* indicates the size of the search window, and *W_ijk_* indicates the weights of similar neighboring pixels in a given window; *W_ijk_* is determined using the following three factors: (1) the spectral distance (*S_ijk_*) between the GF and MODIS data at a given location at *t_k_*, (2) the spatial distance (*D_ijk_*) between a neighboring pixel and the central pixel, and (3) the temporal distance (*T_ijk_*) between the input data and the predicted MODIS data [27].

##### Spatially Downscaling Model Constructed by SSCF

Different scholars have proposed different methods for calculating SSCF by constructing various indexes that are highly correlated with SM. By simplifying the DISPATCH method [17,34], Kim and Hogue proposed the UCLA downscaling method based on the soil wetness index (UCLA-SWI) to construct an SSCF [21]. Peng et al. [22] improved this method by using the vegetation temperature condition index (UCLA-VTCI). Wang et al. [18] proposed a PKU-derived downscaling method based on the temperature vegetation drought index (TVDI). Recently, Wang et al. [35] constructed an SSCF by calculating the reciprocal of the slope between the land surface temperature and net surface shortwave radiation. As suggested in the above-described studies, there is a good correlation between the MPDI and SM [23,24]. This paper, thus, attempts to construct an SSCF based on the MPDI:

(10) $$\mathrm{SSCF}=\frac{1-\mathrm{MPDI}}{1-\bar{\mathrm{MPDI}}}$$

where $\bar{\mathrm{MPDI}}$ is the average value within the CCI SM grid box with a 25 km spatial resolution. The MPDI was developed by introducing the fractional vegetation cover (FVC) to remove vegetation information from mixed pixels [23] as follows:

(11) $$\mathrm{MPDI}=\frac{1}{\left(1-FVC\right)*\sqrt{M^{2}+1}}\left(R_{red}+MR_{nir}-FVC*\left(R_{v,\;red}+MR_{v,nir}\right)\right).$$

(12) $$\mathrm{PDI}=\frac{1}{\sqrt{M^{2}+1}}\left(R_{red}+MR_{nir}\right).\;$$

where PDI indicates the perpendicular drought index [36]; *R_red_* and *R_nir_* represent the surface reflectance of the red band and near-infrared band, respectively; *R_v, red_* and *R_v, nir_* can be determined as known vegetation constants [23]; *M* represents the slope of the soil line in the study area; and FVC is calculated using the dimidiate pixel model based on the normalized difference vegetation index (*NDVI*). The calculation of FVC is expressed as follows:

(13) $$\mathrm{FVC}=(NDVI-NDVI_{soil})/(NDVI_{veg}-NDVI_{soil})$$

where *NDVI_soil_* represents the *NDVI* value of pixels that are completely composed of bare soil, and *NDVI_veg_* represents the *NDVI* value of pixels that are completely covered by vegetation.

Finally, the downscaled CCI SM images could be obtained by the spatially downscaling formula:

(14) $$\mathrm{SM}=\mathrm{SSCF}*\bar{\mathrm{SM}}$$

where SM is the downscaled CCI SM with a 16 m spatial resolution, and $\bar{\mathrm{SM}}$ is the original CCI SM at a 25 km spatial resolution. Additionally, in the actual calculation process, the value of MPDI was normalized, according to the maximum and minimum value of the original CCI image, since MPDI and SM had different value ranges.

#### 2.3.3. Evaluation Methods

The downscaled results were verified by the following three methods: (1) the downscaled data on 1 May, 11 May, 21 May, and 1 June were compared with in situ SM observations using the R, RMSE, slope, average absolute difference (AAD),and other statistical indicators; (2) the original CCI SM images were visually compared with the corresponding downscaled SM data to qualitatively analyze the spatial validity of the downscaling method; and (3) the time series of the original CCI SM data and the downscaled SM data representing 8 in situ stations were compared to analyze the temporal validity of the downscaling method.

## 3. Results

### 3.1. Comparison of Downscaled CCI SM Data with In Situ Observations

**Figure 4:**
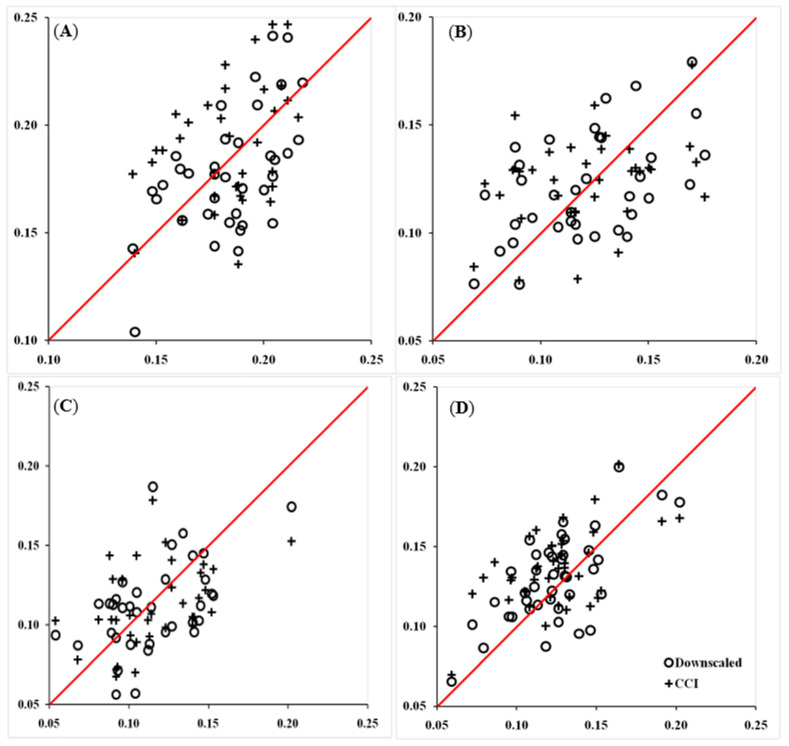
Scatterplot comparisons of in situ SM data (x-axis, cm^3^/cm^3^) versus the downscaled SM data (y-axis, circle-indicated, cm^3^/cm^3^) and the original CCI SM data (y-axis, cross-indicated, cm^3^/cm^3^) on 1 May (**A**), 21 May (**B**), 1 June (**C**), and 11 June (**D**).

Figure 4 shows the scatterplots of in situ SM observations versus the downscaled SM data or the original CCI SM data. The scatterplots representing all dates were concentrated on both sides of the 1:1 line, indicating good consistency between the downscaled SM data and in situ SM data collected on all dates. Compared with the scatterplots between the original CCI SM and in situ SM data, the scatterplots between the downscaled SM data and in situ SM data were more scattered and mostly had larger maximum values and smaller minimum values on all dates. The statistical values represented by these scatterplots and the comparisons among dates are shown in Table 2. All statistical indicators showed that the downscaled SM data were more relative and accurate to in situ SM data compared with the original CCI SM data. The R values ranged from 0.45 to 0.67 for the downscaled SM data versus 0.33 to 0.54 for the original CCI SM data. The RMSE values ranged from 0.023 to 0.031 cm^3^/cm^3^ for the downscaled SM data versus 0.027 to 0.032 cm^3^/cm^3^ for the original CCI SM data.

### 3.2. Visual Comparison of the Downscaled CCI SM Data with the Original CCI SM Data

**Figure 5:**
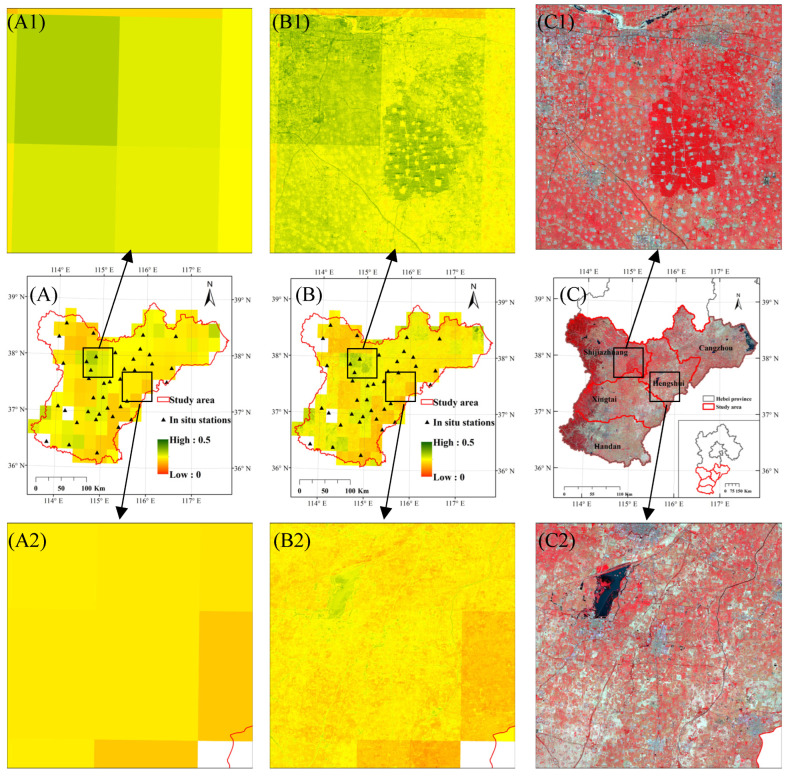
Spatial comparisons of the original CCI SM image (**A**), the downscaled CCI SM image (**B**) on 1 June, and the NIR-red-green composite of a GF image taken on 3 June (**C**) over the study area. The partial magnifications (**A1**–**C1**) showed the details of a small area of high SM values. The partial magnifications (**A2**–**C2**) showed the details of a small area of low SM values.

Figure 5 shows maps comparing the original CCI SM images with the downscaled SM images on 1 June and the reflectance NIR-red-green composites of the GF image on 3 June. On the whole, the spatial distribution of SM in both showed a certain correlation with ground objects. Except for a few areas in the central and northeastern regions where the SM was higher due to the presence of vegetation or water bodies, the SM was lower in most areas in the northwestern, eastern, and southeastern regions, where there was less vegetation. However, by comparing the local magnified images, it can be found that the downscaled SM image had clearer outlines, richer textures, higher spatial consistency with the ground objects, and can describe the spatial distribution of SM in more detail, compared with the original CCI SM image. For example, in an area with high SM (Figure 5(A1)), some low-SM-value objects, such as roads and construction areas, can be clearly distinguished from the downscaled SM image (Figure 5(B1)); in an area with low SM (Figure 5(A2)), a small number of areas with high SM can be found from the downscaled SM image (Figure 5(B2)).

### 3.3. Evaluation of Downscaling Methods Using Time Series In Situ Observations

**Figure 6:**
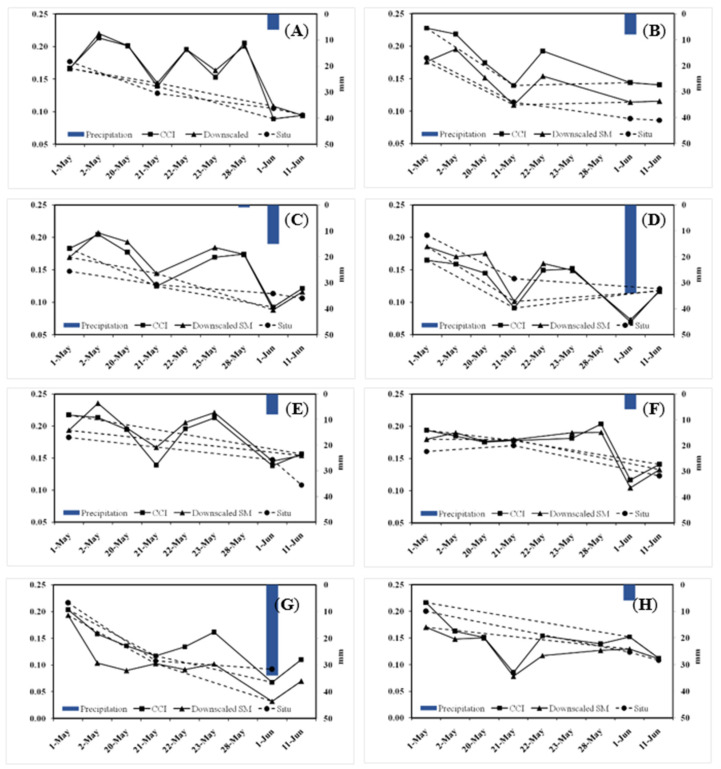
Comparisons of the time series curves of the original CCI SM data (cm^3^/cm^3^), in situ SM data (cm^3^/cm^3^), and downscaled SM data (cm^3^/cm^3^) at eight sites (**A**–**H**), respectively, above three valid in situ observations selected). The three or four values of in situ SM and the corresponding original CCI SM and the downscaled SM are connected by dotted lines, respectively. The nine values of the original CCI SM and the corresponding downscaled SMare connected by solid lines. The corresponding precipitation values are presented in bar charts.

Figure 6 shows the timeseries curves of the original CCI SM data, the downscaled SM data, and in situ SM at eight different sites (above three valid in situ observations selected). The values of each site decreased gradually with the time during this period. The corresponding original CCI SM curve and the downscaled SM curves were in good agreement with the in situ SM curve on the whole trend (dotted lines in Figure 6). Moreover, compared with the in situ curve, more details in the continuous dynamic changes of SM were described in the original CCI SM curve or the downscaled SM curve at each site, because of the higher observation time frequency (nine values vs. three or four values). At most points of time (e.g., 1 May, 21 May, 1 June, and 11 June in Figure 6B; 1 May and 21 May in Figure 6D), the downscaled SM values were closer to in situ SM values. At a few points of time (e.g., 1 May and1 June in Figure 6G), the original CCI SM values were closer to in situ SM values. However, the quantitative statistical results showed (Table 3) that compared with the original CCI data, the overall error between the downscaled data and in situ data was smaller. The average error of downscaled SM data at almost all points (except point 7) was smaller (Table 3). The average error of downscaled SM data at all four time points was smaller (Table 3). Therefore, more accurately dynamic changes of SM over time could be reflected by the downscaled SM curve.

## 4. Discussion

### 4.1. Comparison of the Precision of Spatial Downscaling Algorithms

The R and RMSE were the key indexes for evaluating spatially downscaling algorithms. These values were a little different from the results of relevant past research studies [20,22,37,38]. The R values were a little lower than that those in Abowarda et al.’s study [20] and Wei et al.’s study [38], which were, respectively, 0.56 and 0.62. A similar result was shown in a study by Peng et al. [22] (i.e., R ranged from 0.6 to 0.75) and Colliander et al.’s research [37] (i.e., R was about 0.61). This is an interesting phenomenon, which may be related to the spatial resolution ratio between the original CCI SM data and the downscaled SM data. As the ratio increases, the relevance may be less between the downscaled data and in situ data. However, the mean values of the RMSE were significantly lower than that in Abowarda et al.’s study [20] (i.e., RMSE was about 0.07 cm^3^/cm^3^); Wei et al.’s study [38] (i.e., RMSE was about 0.08 cm^3^/cm^3^); Peng et al.’s study [22], where the RMSDs ranged from 0.07 to 0.1 cm^3^/cm^3^); and Colliander et al.’s research [37] (i.e., the RMSD was about 0.05 cm^3^/cm^3^). Therefore, considering the same order of magnitude of R values and significantly lower RMSE values, the proposed method had the highest accuracy.

### 4.2. Spatial and Temporal Improvements for the Original CCI Data

The validation results showed that the accuracy of the downscaled SM data was improved in both spatial and temporal dimensions. Compared with the original CCI SM data, the downscaled SM data could better describe the spatial distribution and temporal dynamic changes of SM. Analysis from the spatial dimension: the original CCI SM reflected the average state of water content of various ground objects within the whole 25 kmpixel area. However, because the downscaled SM pixels were assigned different values according to the characteristics of water content of the ground objects, bigger fluctuations and better spatial correlations with the ground objects were shown exactly (Figure 4), and the spatial details of SM could be expressed more precisely and accurately (Figure 5 and Table 2). Analysis from the temporal dimension: the SM value was the value of dynamic change with time. With a low spatial resolution, the time series fluctuation of original CCI data tended to be “flat” due to the average effect of mixed pixels. The downscaled pixels became relatively “pure”. Therefore, timeseries fluctuations could better reflect the real situation of water content in the pixel (Figure 6 and Table 3).

### 4.3. Characteristics of the Proposed Method

(1) SSCF constructed by MPDI. Referring to physical model-based methods in principle, the spatially downscaling method proposed in this paper utilized the MPDI, which has a strong correlation with SM, to construct SSCF and subsequently downscale the original SM products. MPDI data have the following two important characteristics: first, its value is calculated from the surface reflectance, which usually has a high spatial resolution, and can show a better correlation with the ground objects; second, its value is closely related to SM, and the fluctuation of MPDI time series can better reflect the dynamic change of SM. The above two characteristics of MPDI data were conducive to the improvement of the original CCI data in spatial and temporal dimensions, which supported the method proposed in this paper, which has the following advantages.
(2) Finer spatial resolution. The various downscaling methods utilized in the past usually require land surface temperature data as input data [21,39]. However, land surface temperature data usually have low spatial resolutions, generally above 1 km [38,40], directly leading to traditional downscaling algorithms having difficulty obtaining downscaled results with a high spatial resolution. However, the proposed method needs only surface reflectance data, which usually have higher spatial resolutions, as the input data, so the proposed method can obtain downscaled results with higher spatial resolutions (e.g., 16 m).
(3) High temporal resolution maintained. Higher spatial resolution is usually at the expense of temporal resolution, because higher spatial resolution data are often acquired by longer intervals and disturbed by more interference of clouds and fog. This “*spatiotemporal contradiction*” will lead to the low temporal resolution of downscaled data, and then cause a decrease in SM monitoring frequency. To solve this problem, a spatiotemporal data fusion algorithm (e.g., ESTARFM) was introduced in this paper to build a surface reflectance dataset with a high spatial and temporal resolution to ensure that not only the spatial fineness can be improved, but also the high temporal resolution can be maintained, thus better serving practical production applications.
(4) Technical process simple and easy to implement. Some studies have effectively downscaled CCI SM products to a 30 m spatial resolution by combining a spatiotemporal data fusion algorithm with a random forest algorithm [12,32]. However, such methods require more input data than the proposed method. Additionally, their calculation processes are more complex. The technical process followed in this paper was divided into three steps. Further, the calculation process of each step was relatively simple. Compared with other downscaling methods, the proposed method not only requires fewer original data types but also has a relatively simple technical process. Therefore, the method proposed in this paper is easy to implement and has good practicability.

## 5. Conclusions

SM is an important surface parameter. At present, many global-scale SM products have been created based on remote sensing technologies. However, existing SM products have low spatial resolution. Therefore, it is urgent to develop corresponding spatially downscaling algorithms to improve the spatial resolutions of existing SM products. In this paper, a new spatially downscaling method was proposed. (1) First, a surface reflectance dataset with high spatial and temporal resolution was constructed based on the ESTARFM algorithm. (2) Then, an SSCF dataset was constructed by a MPDI dataset and an aggregated MPDI dataset, both calculated by the surface reflectance dataset. (3) Finally, a downscaled SM dataset was obtained, combining the SSCF dataset and the original SM product.

In this study, five cities in southern Hebei Province were selected as the study area. TwoGF6 images and nine MOD09GA images were used to downscale nine CCI SM images obtained from May to June 2019. A total of 151 in situ SM observations collected from ground-monitoring stations on four dates were used to verify the results. The correlation coefficients between the downscaled SM data and the in situ SM data ranged from 0.45 to 0.67, and the RMSE values ranged from 0.023to 0.031 cm^3^/cm^3^. In addition, the spatial fineness of the downscaled SM data was significantly higher than that of the original CCI SM data. Therefore, the proposed method can not only effectively downscale CCI SM products (from 25 km to 16 m spatial resolution) but also maintain a high temporal resolution. In addition, compared with other traditional downscaling methods, the proposed method has the advantages of a higher spatial fineness and an easy implementation process. The research results reported in this paper will be beneficial for obtaining more precise SM data for farmland management.

## Figures and Tables

**Table 1:** Data used in this research.

GF6		MOD09GA		ESA CCI		In Situ	
Date	Usage	Date	Usage	Date	Usage	Date	Usage
		1 May	ESTARFM algorithm input	1 May	Original data for downscaling	1 May	Validation
2 May	ESTARFM algorithm input	2 May	ESTARFM algorithm input	2 May	Original data for downscaling		
		20 May	ESTARFM algorithm input	20 May	Original data for downscaling		
		21 May	ESTARFM algorithm input	21 May	Original data for downscaling	21 May	Validation
		22 May	ESTARFM algorithm input	22 May	Original data for downscaling		
		23 May	ESTARFM algorithm input	23 May	Original data for downscaling		
		28 May	ESTARFM algorithm input	28 May	Original data for downscaling		
3 June	ESTARFM algorithm input	1 June	ESTARFM algorithm input	1 June	Original data for downscaling	1 June	Validation
		11 June	ESTARFM algorithm input	11 June	Original data for downscaling	11 June	Validation

**Table 2:** Statistical comparisons of RMSE, correlation coefficient, and slope indicators between the original CCI SM data or the downscaled CCI SM data and in situ SM data on 1 May, 21 May, 1 June, and 11 June.

Date	N	CCI			Downscaled		
		RMSE (cm^3^/cm^3^)	R	Slope	RMSE (cm^3^/cm^3^)	R	Slope
1 May	37	0.029	0.43	0.61	0.025	0.57	0.76
21 May	36	0.029	0.33	0.24	0.026	0.51	0.44
1 June	37	0.032	0.35	0.32	0.031	0.45	0.48
11 June	41	0.027	0.54	0.36	0.023	0.67	0.66

**Table 3:** Statistical comparisons of average absolute difference (AAD) values between the original CCI SM data or the downscaled CCI SM data and in situ SM dataon1 May, 21 May, 1 June, and 11 June. The lower AAD values are noted in bold.

Sites No.	Category	1 May (cm^3^/cm^3^)	21 May (cm^3^/cm^3^)	1 June (cm^3^/cm^3^)	11 June (cm^3^/cm^3^)	Mean (cm^3^/cm^3^)
1	CCI	**0.010**	**0.011**	0.016	**0.000**	0.009
	Downscaled	0.011	0.016	**0.003**	0.001	**0.008**
2	CCI	0.046	0.026	0.056	0.054	0.045
	Downscaled	**0.006**	**0.004**	**0.026**	**0.029**	**0.016**
3	CCI	0.035	**0.002**	**0.020**	0.016	0.018
	Downscaled	**0.021**	0.018	0.025	**0.010**	**0.018**
4	CCI	0.039	0.045	-	**0.003**	0.029
	Downscaled	**0.017**	**0.035**	-	0.004	**0.019**
5	CCI	0.035	-	0.009	0.049	0.031
	Downscaled	**0.012**	-	**0.002**	**0.046**	**0.020**
6	CCI	0.033	**0.008**	-	0.018	0.020
	Downscaled	**0.019**	0.009	-	**0.010**	**0.012**
7	CCI	**0.012**	0.009	**0.024**	-	**0.015**
	Downscaled	0.023	**0.005**	0.060	-	0.029
8	CCI	**0.017**	-	0.029	0.004	0.017
	Downscaled	0.030	-	**0.006**	**0.003**	**0.013**
Mean	CCI	0.028	0.017	0.026	0.020	0.023
	Downscaled	**0.017**	**0.015**	**0.020**	**0.015**	**0.017**

## Data Availability

The GF data used in the research was freely available in the online portal of China Centre For Resources Satellite Data and Application website. The MODIS data was freely available in the online portal of USGS-NASA website. And the CCI data was freely available in the online portal of ESA website.
